# Neonatal Respiratory Depression Associated With Maternal Oxycodone Use During Breastfeeding

**DOI:** 10.7759/cureus.105882

**Published:** 2026-03-26

**Authors:** Madison L Wallace, Renee Himmelbaum

**Affiliations:** 1 Obstetrics and Gynecology, AU/UGA Medical Partnership, Athens, USA; 2 Pediatrics, AU/UGA Medical Partnership, Athens, USA

**Keywords:** breastfeeding, breastmilk, drugs in breast milk, maternal opioid use, neonatal apnea, neonatal respiratory depression, oxycodone, oxycodone breastmilk

## Abstract

We report a case of a term male neonate, born via repeat cesarean section (CS), who developed transient tachypnea of the newborn (TTN) shortly after birth, followed by two episodes of bradycardia with desaturation and apnea/bradycardia/desaturation on days of life (DOL) three and four, respectively, temporally linked to maternal oxycodone use during breastfeeding. The infant was transitioned from maternal breast milk to donor milk until the mother’s oxycodone dose was below 40 mg/day. This report highlights the importance of careful monitoring for respiratory depression and bradycardia in neonates exposed to maternal opioids through breast milk, particularly at doses exceeding recommended levels.

## Introduction

Oxycodone is frequently prescribed to manage moderate to severe postoperative pain after cesarean section (CS), valued for its high oral bioavailability and well-established efficacy in multimodal analgesia regimens [1]. However, its use in lactating women raises concern due to excretion into breast milk and the resulting potential for neonatal exposure [2,3]. Neonates are particularly vulnerable to opioid-related toxicity because of their immature hepatic metabolism and renal clearance, which can prolong drug elimination and enhance central nervous system (CNS) effects [3,4]. Oxycodone is metabolized to active compounds, including oxymorphone, which has a much higher affinity for μ-opioid receptors and greater potency than the parent drug [5]. Even small concentrations in breast milk can result in clinically significant exposure for neonates, especially in the early postnatal period when hepatic enzyme activity is limited [4,5]. This case report adds to the growing literature suggesting a potential association between high-dose maternal oxycodone use and neonatal respiratory events, highlighting the need for further investigation and more cautious prescribing in lactating patients.

## Case presentation

A male neonate was born via repeat cesarean section at 37 weeks and four days of gestation to a G2P1 mother in her early 30s after she presented to the hospital in early active labor. The mother had not experienced any complications during this pregnancy and received routine prenatal care throughout. She had taken a prenatal vitamin with iron, Zyrtec, and a short course of antibiotics for a sinus infection while pregnant. She had a history of marijuana gummy use before pregnancy, but stopped using once she knew she was pregnant and did not resume use while breastfeeding. Syphilis, HIV, group B streptococcus, hepatitis B, hepatitis C, chlamydia, and gonorrhea screenings at the time of delivery were negative.

**Figure 1:**
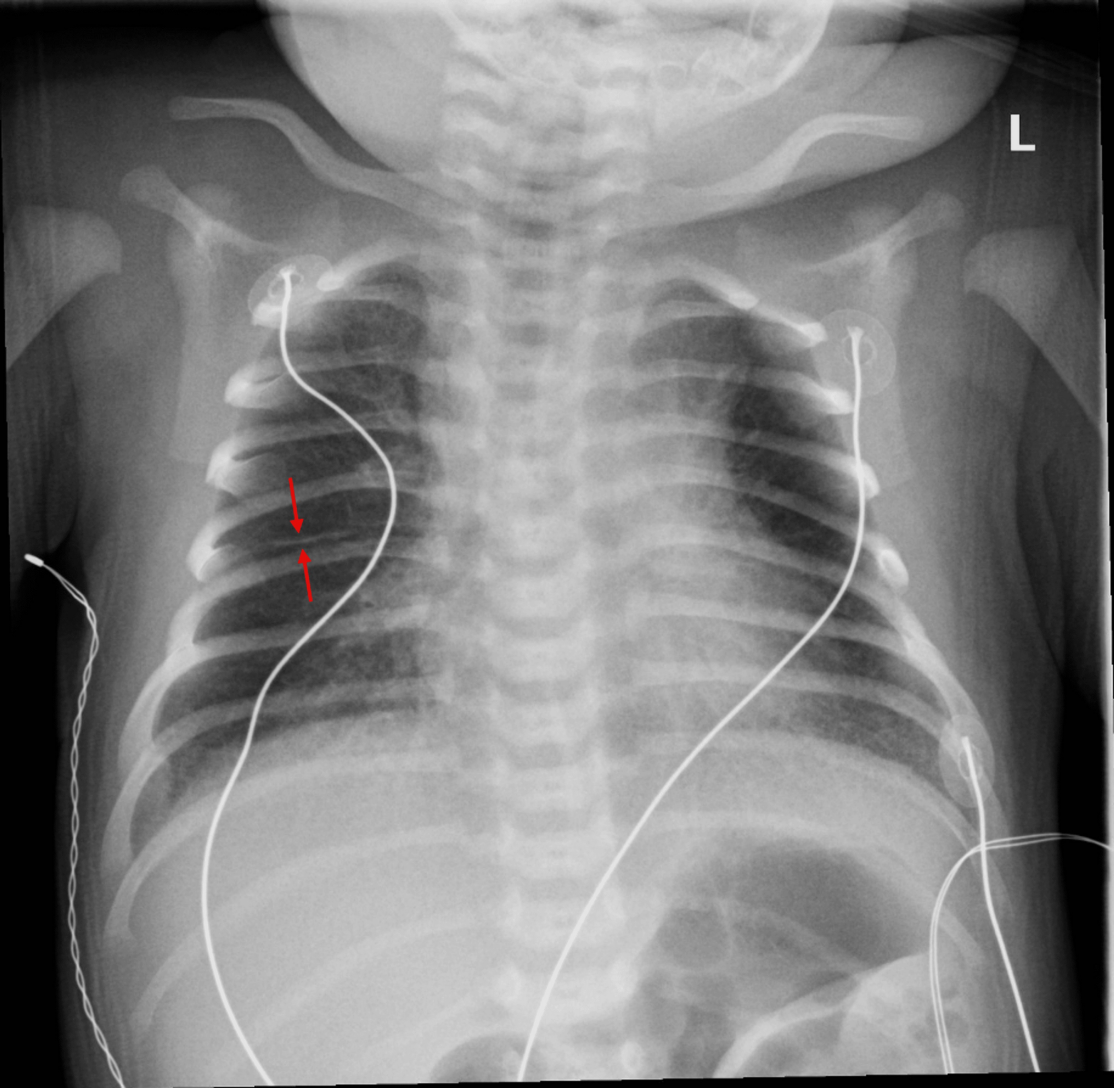
Neonatal chest radiograph Chest radiograph was ordered after continued neonatal respiratory distress and revealed bilateral perihilar streaking extending out into the lung fields with fluid in the minor fissure (red arrows)

The neonate weighed 3.76 kg at birth and measured 51 cm long with a head circumference of 35.5 cm. Apgar scores were 8 and 9 at one and five minutes, respectively. However, he required neonatal intensive care unit (NICU) admission for continued respiratory distress, later determined to be secondary to transient tachypnea of the newborn (TTN) with chest radiograph demonstrating perihilar streaking and fluid in the minor fissure (Figure 1).

The neonate was placed on nasal continuous positive airway pressure (5 cmH_2_O; FiO_2_: 0.21) from days of life (DOL) zero to two with supplemental oxygen via nasal cannula (1 lpm; FiO_2_: 0.25) on DOL zero and was successfully weaned to room air on DOL two (Table 1). While in the NICU, he was on gavage feeds of formula and maternal breast milk every three hours. After his respiratory distress resolved, he was transitioned to only maternal breast milk every three hours.

**Table 1:** Neonatal capillary blood gas trends during early NICU course Capillary blood gases obtained on April 18, 2025 (DOL one) and April 19, 2025 (DOL two) demonstrate initial respiratory acidosis with elevated pCO₂ on 4/18, consistent with respiratory distress shortly after birth requiring NICU admission and respiratory support with bubble CPAP. Serum bicarbonate levels were elevated on both 4/18 and 4/19, reflecting a compensatory metabolic response. With continued CPAP support from DOL zero to two, capillary blood gases showed improvement in pCO₂ by 4/19, corresponding with clinical improvement. The infant was successfully transitioned to room air on 4/19 (DOL two) following stabilization NICU: neonatal intensive care unit; pCO₂: partial pressure of carbon dioxide; pO₂: partial pressure of oxygen; HCO₃: bicarbonate; base excess: metabolic component of acid–base balance; SpO₂: peripheral oxygen saturation; FiO₂: fraction of inspired oxygen; CPAP: continuous positive airway pressure; EPAP: expiratory positive airway pressure

Variable	April 18, 2025, 0:03	April 19, 2025, 5:43	Units	Reference range
pH	7.307	7.37		7.30 - 7.49
pCO_2_	53.2 (H)	46.9	mmHg	35 - 49
pO_2_	33	38	mmHg	
HCO_3_	26.6 (H)	27.1 (H)	mmol/L	22 - 26
Base excess	-0.6	1.2		-2 - 2
SpO_2_	94	95		
FIO_2_ RT	25	21		
O_2_ device	Bubble CPAP	Bubble CPAP		
Flow rate	10	10		
EPAP	6	5		
Sample site	L heel	R heel		

**Figure 2:**
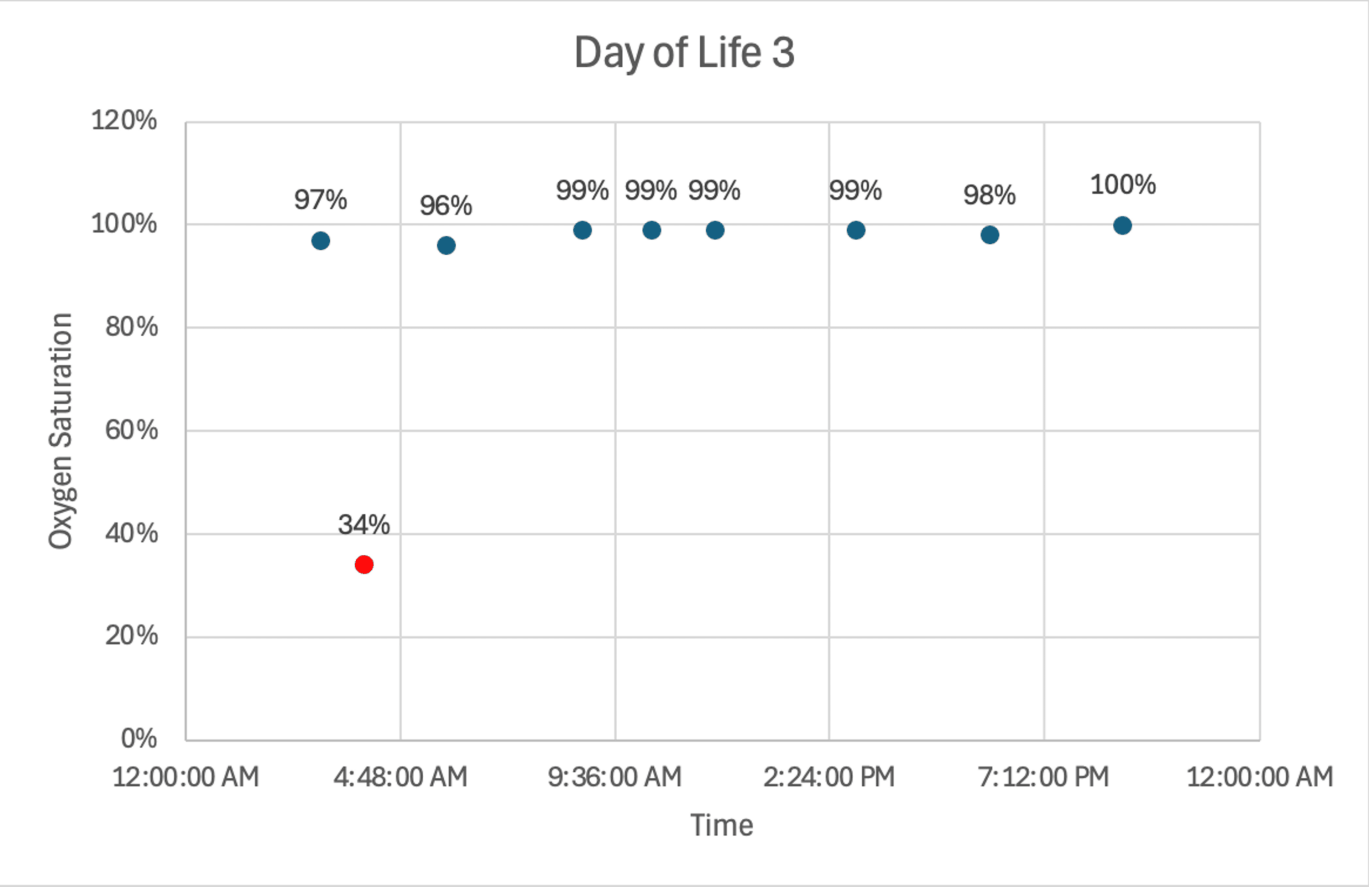
Oxygen saturation trends on DOL three following maternal oxycodone exposure via breast milk Continuous spot oxygen saturation measurements on DOL three demonstrate overall stable saturations in the high 90s, with a single acute episode of bradycardia and severe desaturation (heart rate: 64 beats/min, oxygen saturation: 34%) occurring during stooling (red data point) DOL: day of Life

**Figure 3:**
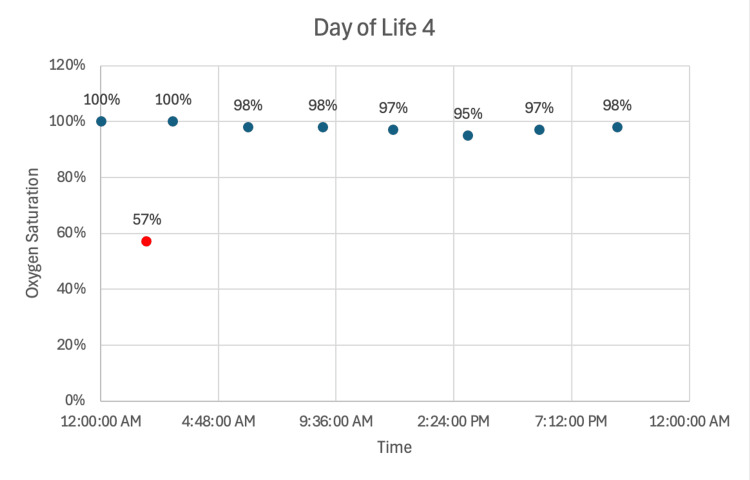
Oxygen saturation trends on DOL four with recurrent cardiorespiratory event prompting feeding modification Oxygen saturation measurements on DOL four again show predominantly stable values in the mid-to-high 90s, with a recurrent episode of bradycardia and desaturation (heart rate: 69 beats/min, oxygen saturation: 57%) associated with an apneic event in the early morning hours (red data point) DOL: day of Life

Notably, the mother was prescribed 60 mg/day of oxycodone via patient-directed analgesia on DOL two. She continued to breastfeed through either direct nursing or expressed milk. On DOL three, the neonate experienced an episode of bradycardia and desaturation (heart rate: 64, oxygen saturation: 34%) while stooling, which recurred the following morning along with an associated apneic event (heart rate: 69, oxygen saturation: 57%) (Figures 2, 3). As a result, maternal breast milk was held, and donor milk and formula were initiated. The mother was instructed to discard expressed milk until her oxycodone dose decreased to 40 mg/day. She ultimately chose to discontinue oxycodone and delay breastfeeding until 48 hours after her last dose. No further apneic events occurred following the transition to donor milk and formula. The neonate was discharged home on DOL seven, weighing 3.41 kg, on a formula diet supplemented with poly-vi-sol vitamin drops with iron.

The neonate appeared well at his newborn well-child check on DOL eight. He weighed 3.4 kg, representing a 9.5% decrease from birth weight. Earlier that morning, his mother had reintroduced breast milk into his diet, as it had been 48 hours since her last dose of oxycodone. He tolerated the maternal breast milk well and had no recurrent episodes of respiratory distress. Review of systems and physical examination were negative for any concerning findings. The patient appeared to be growing and developing appropriately without signs of illness. Anticipatory guidance was provided to both parents, and a follow-up appointment was scheduled two days later to closely monitor for excessive weight loss.

## Discussion

Pharmacokinetic studies show that the milk-to-plasma ratio of oxycodone often exceeds 3.0, with modeled neonatal exposures reaching up to 10% of a therapeutic infant dose in worst-case scenarios [1]. In a retrospective study, Lam et al. reported a 20.1% incidence of CNS depression in infants breastfed by mothers taking oxycodone, compared to only 0.5% in those exposed to acetaminophen alone (odds ratio (OR): 46.16) [5]. Although oxycodone has been used widely and generally safely, adverse events including sedation, bradycardia, and respiratory depression have been reported, particularly with high maternal doses or concurrent CYP3A4 or CYP2D6 inhibition [6,7]. The LactMed database recommends limiting maternal oxycodone to 60 mg/day for no more than two to three days, especially in exclusively breastfed infants under two months old [2]. Other clinical guidelines, including those from the King Edward Memorial Hospital (KEMH), recommend even stricter thresholds of no more than 40 mg/day and for no longer than three days [3].

The Society for Obstetric Anesthesia and Perinatology (SOAP) acknowledges the risks of neonatal sedation but recommends caution in completely discontinuing oxycodone due to the limited clinical use and pharmacologic drawbacks of alternative opioids such as morphine and hydromorphone. SOAP supports multimodal analgesia and recommends that oxycodone be used only as rescue therapy at the lowest effective dose for the shortest possible duration [8]. Importantly, a recent cross-sectional study by Ahmadzai et al. found that mothers tended to underreport suspected adverse drug reactions (ADRs) in their breastfed infants, with opioids among the most frequently implicated medications [9]. These findings highlight the need for greater awareness and monitoring of opioid-related effects in neonates, especially during the early postnatal period when breastfeeding is often most frequent and maternal dosing may be highest.

## Conclusions

This report demonstrates the potential risk of neonatal opioid exposure via breast milk, particularly in the early neonatal period when hepatic and renal metabolism are immature. Oxycodone is excreted in breast milk, and while levels are generally low, high maternal doses, as in this case, may result in clinically significant neonatal exposure. Apnea and bradycardia in neonates have multifactorial causes, but the temporal association with maternal high-dose oxycodone use, resolution after stopping breast milk, and absence of other risk factors in this case report suggest a possible link. While TTN explains the initial respiratory distress, the subsequent apnea events, particularly on DOL four, raise concern for opioid-related central respiratory depression. Current guidelines caution against high-dose opioid use in breastfeeding mothers and recommend using the lowest effective dose for the shortest duration.
